# Surgical management of benign tumors of the parotid gland: the advantages of extracapsular dissection compared to traditional surgical techniques

**DOI:** 10.3389/fsurg.2024.1415485

**Published:** 2025-01-15

**Authors:** Antonio Zanghì, Andrea Cavallaro, Martine Marchi, Marcello Marchi, Luigi La Via, Filippo Sanfilippo, Alessandro Cappellani, Simone Di Majo

**Affiliations:** ^1^ Department of General Surgery and Medical-Surgical Specialties, Chief ChiSMaCoTA Research Center, AOU Policlinico “G. Rodolico - San Marco”, Catania, Italy; ^2^ General Surgery III, Department of General Surgery and Medical-Surgical Specialties, University of Catania, AOU Policlinico “G. Rodolico - San Marco”, Catania, Italy; ^3^ Maxillofacial, Plastic and Reconstructive Surgery Unit, Centro Clinico Diagnostico G. B. Morgagni, Catania, Italy; ^4^ Department of Anaesthesia and Intensive Care, AOU Policlinico “G. Rodolico - San Marco”, Catania, Italy; ^5^ School of Anaesthesia and Intensive Care, University of Catania, AOU Policlinico “G. Rodolico - San Marco”, Catania, Italy

**Keywords:** salivary gland, tumor, surgery, extracapsular dissection, parotid gland, benign neoplasia

## Abstract

**Introduction:**

Salivary gland tumors represent only 3%–6% of all head and neck neoplasms, and approximately 70% of these tumors are located in the parotid gland. Most of these tumors are found in the more abundant superficial portion of the parotid gland, lateral to the facial nerve (FN). For many years, the location of the facial nerve between the superficial and deep segments of the parotid gland hindered adequate tumor extirpation. Several surgical options are available for the treatment of benign tumors in the parotid gland, but there remains no universal agreement on what the optimal surgical treatment is. In the early twentieth century, tumor enucleation was the standard treatment for parotid tumors to preserve the facial nerve, but high recurrence rates were the main downside of this procedure. To improve the outcome, superficial parotidectomy (SP) was implemented, which involves excision of the entire lateral segment of the parotid gland, superficial to the facial nerve. However, this surgical procedure may lead to severe postoperative complications, including facial nerve paralysis, in a significant number of patients. In recent years, more gland-preserving techniques were developed to reduce complication rates and improve the safety of procedures and patients' satisfaction, without increasing the risk of recurrence.

**Materials and method:**

This study compares our surgical experience with extracapsular dissection gland-sparing surgery (ECD) to traditional superficial parotidectomy in 56 patients who underwent surgery performed by the same surgical team.

**Results:**

The superiority of ECD procedures compared to SP procedures was shown as far as total complication rates are concerned. In this case, Fisher's exact test statistic value was 0.0043 (significant at *P* < 0.05).

**Conclusion:**

ECD should be applied in properly selected cases and further prospective studies are needed to clarify the optimal indications.

## 1 Introduction

Salivary gland tumors represent only 3%–6% of all head and neck neoplasms, and approximately 70% of these tumors are located in the parotid gland. Parotid gland tumors arise from the same stem-cell differentiation pathways as normal salivary gland tissues. Although the etiology of benign salivary tumors remains unknown, factors such as smoking, radiation, viruses, genetics, and trauma have been associated with their development.

There is a correlation between radiation exposure and salivary gland tumors, with 50% of radiation-induced tumors being pleomorphic adenomas (PAs) (1).

Viral infections such as human papillomavirus (HPV) types 6 and 11 may increase the risk of developing ductal papilloma (inverted type) (2), whereas in the case of lymphadenoma (LA), IgG4-positive plasma cells may indicate that immunomodulation plays a role in its etiology (3).

The association between Warthin’s tumor (WT) and tobacco is widely known (4, 5): multifocality and bilaterality are especially important in heavy smokers (6).

The role of genetics is shown; hence, pleomorphic adenoma gene 1 (PLGA1) is activated by chromosomal translocation involving 8q12, while high-mobility group AT-hook 2 (HMGA2) is activated by the rearrangement at 12q13-15. Both genes are highly specific for pleomorphic adenomas and carcinoma ex-pleomorphic adenomas (7).

The semi-pluripotent bicellular reserve cell theory, which is the most widely accepted theory of salivary gland tumor histogenesis, suggests that basal cells of the excretory ducts and progenitor cells of the intercalated ducts have the potential for cellular division and tumor formation. In contrast, the acinar units and striated ducts, which are terminally differentiated, cannot evolve into neoplasms.

About 70% of salivary gland tumors are pleomorphic adenomas (PAs).

PAs are mostly located in the superficial portion of the parotid gland, while only 10% are in the deep lobe. These tumors may be synchronous or metachronous with other tumors but are usually solitary capsulated lesions and are rarely multifocal.

Warthin's tumor (WT) is the second most common benign tumor of the salivary glands and the most common synchronous multiple tumor. Tabagism is known to be strongly associated with multifocality and bilaterality. WT presents as a well-encapsulated tumor, usually in the caudal pole of the parotid gland: malignant transformation is rare (1).

Other benign histotypes are oncocytoma (approximately 2% of all salivary gland neoplasms), cystadenoma, myoepithelioma (MYO), sebaceous adenoma, basal cell adenoma, lymphadenoma (LA), sialadenoma papilliferum, canalicular adenoma, ductal papilloma (intraductal type and inverted type), sclerosing polycystic adenoma (SPA), keratocystoma, intercalated duct adenoma (IDA), and striated duct adenoma (SDA) (8).

## 2 Surgical techniques

The most recent classification system for parotidectomy surgeries was proposed by the European Salivary Gland Society (ESGS) using only two terms: “parotidectomy” and “extracapsular dissection” (ECD).

In this classification, the parotid parenchyma is subdivided into five levels. The concept of superior and inferior is established considering where the division of the main trunk of the facial nerve (FN) occurs. The superior level corresponds to the area traversed by the temporofacial branch and the inferior level to the path of the cervicofacial branch.

• I (lateral superior)
• II (lateral inferior)
• III (deep inferior)
• IV (deep superior)
• V (accessory)

Based on the number and type of structures removed (glandular or non-glandular), the extent of resection during parotidectomy is thoroughly defined. ECD includes instead level I or level II only to indicate tumor location, since this technique does not involve removal of the parotid gland up to one level. The removal of one level combined with facial nerve dissection goes beyond the definition of ECD and is named parotidectomy (9).

**Extracapsular dissection (ECD)** is a surgical technique that avoids facial nerve dissection and removes less than one level of the gland. It is usually performed for small tumors. With the increasing size of the tumor and proximity to the facial nerve, surgical dissection becomes more challenging, and the risk of facial nerve injury rises.

Surgeons can perform ECD on tumors larger than 2.5 cm in the caudal part of the gland with some contact to the cervicofacial branch of the facial nerve (ESGS level II), tumors of the cranial superficial part of the gland with contact to the temporal branch of the facial nerve (ESGS level I) and small tumors near Stensen's duct (ESGS level V). A limited incision caudal to the ear lobule or briefly extending to the preauricular area can be performed (9, 10).

**Partial superficial parotidectomy (PSP)** is indicated for tumors in the caudal part of the gland (ESGS level II) with contact to the main trunk of the facial nerve and large tumors (>4 cm) of the caudal part of the gland (ESGS level II) with contact to the main trunk and the cervicofacial branch of the facial nerve (11, 12).

**Superficial parotidectomy (SP):** Tumors of the cranial part of the parotid gland often require superficial parotidectomy (SP) for successful removal. SP can be performed (e.g., in cases where extracapsular dissection does not ensure a complete excision) for dissection of every benign tumor of the superficial part of the parotid gland (ESGS levels I and II, but also when the tumor is in level III). Superficial parotidectomy (SP) is typically reserved for medium-sized tumors with contact to both main divisions of the facial nerve: the cervicofacial and temporofacial branches. It can also be performed to extirpate large tumors of the cranial part of the gland (ESGS level I) with contact to the temporal branch and extension up to the main trunk of the facial nerve, as well as large tumors with contact to multiple branches of the nerve not extended to the deep lobe of the gland (9, 12).

**Deep lobe parotidectomy** is the preferred approach for small deep lobe tumors that lie under the facial nerve and do not reach the parapharyngeal space (PPS). These tumors are excised using a standard transparotid approach. Deep lobe parotid tumors that reach the PPS instead are extirpated using several surgical strategies. The isolated transcervical approach involves a cervical incision and dissection of the digastric muscle reaching the hyoid bone. The submandibular and parotid glands are then retracted to access the parapharyngeal space (PPS) medial to the carotid artery. The transcervical–transparotid combined access is the preferred method for managing tumors in the PPS, allowing for excision through extracapsular dissection of the deep lobe of the gland (13).

**Total parotidectomy** is indicated for the treatment of malignant tumors. In benign cases, TP is usually preserved for tumors of the deep lobe with contact to the facial nerve (ESGS level IV), dumbbell tumors beneath the facial nerve that herniate into the stylomandibular tunnel, tumors of the superficial lobe extending into the deep lobe, and multiple tumors of the gland (9). The main downsides include a higher incidence of temporary and permanent facial nerve palsy, Frey's syndrome, and aesthetic deformities.

## 3 Complications in parotid surgery

**Injury to the facial nerve** is the most frequent complication of parotid surgery, resulting in either permanent or temporary facial paralysis despite the nerve being preserved. The incidence of iatrogenic transient facial nerve weakness ranges from 10% to 68%, with most cases resolving within 6 months—90% of cases resolve within 1 month (6). However, in some cases, it can last up to 18 months. This condition is caused by a stretch, compression or ischemic injury to the nerve. The rate of permanent facial nerve palsy rate after surgery ranges from 0% to 19%, and the severity of permanent impairment and disabilities varies depending on which nerve branches are affected.

Palsy rate is generally higher in patients with large or deep lobe tumors (14) and is somewhat proportional to the length of time the nerve is exposed at the time of surgery (15). Diabetes, older age, malignancy, and revision surgery are also known to increase the probability of nerve dysfunction.

Clinical assessment of facial nerve function involves evaluating the symmetry of the resting face, muscle movement, and secondary features such as synkinesis. Facial paralysis disabilities include oral commissure and oral cavity incompetence, as well as nasal valve stenosis with obstruction of the nasal airway (16). More serious consequences may affect the eye, with lagophthalmos and ectropion causing excessive dryness and increasing the risk of exposure to keratitis and bacterial infections (17). Facial nerve impairment is assessed by the House–Brackmann (HB) grading system on a scale of 0–6 by investigating three areas (mouth, eye, and forehead). However, the HB grading system has limitations in detecting synkinesis (18–20).

**Frey's syndrome** (gustatory hyperhidrosis or sweating) is caused by an abnormal reinnervation following injury to the auriculotemporal nerve. The parasympathetic fibers of this nerve stimulate saliva, whereas its sympathetic fibers innervate the sweat glands of the face and scalp. Infrared thermography and Minor's starch-iodine sweating test are used to objectively evaluate the condition (21, 22) Frey's syndrome can also occur during other surgical manipulation or trauma to the parotid region, temporomandibular joint (TMJ) injury, infection, and obstetric trauma (9). Tumor size (≥4) and mass volume are the only statistically significant predictors of Frey’s syndrome development (23). To prevent this condition, increasing the thickness of the skin flap raised over the parotid gland during surgery can help protect the sweat glands and their nerve fibers from exposure. Similarly, a physical barrier, such as a superficial musculoaponeurotic system (SMAS) flap or temporoparietal fascia (TPF) flap can be used, which can also improve facial deformity issues after surgery. Medical therapies for Frey's syndrome include local injection of botulinum toxin, which can reduce gustatory sweating within 48–72 h.

**Parotid fistula and sialocele:** A communication between the skin and a salivary duct or gland, through which saliva is discharged (fistula) or a collection of saliva (sialocele) can gather under the musculocutaneous flap or drain through the wound, has been rarely described (24).

**Parotid tumor recurrence** after previous surgery: The presence of pseudopodia or an incomplete pseudocapsule associated with intraoperative enucleation or tumor spillage are considered main risk factors for tumor recurrence. Recurrent PAs are multicentric and require a more aggressive surgical approach, such as total parotidectomy or radiation therapy (RT). Similarly, incomplete excision of all tumor foci of WT may lead to neoplasm reappearance, but WT may also display new foci as a metachronous occurrence. Other major salivary gland tumors, namely, oncocytoma, myoepithelioma, canalicular adenoma, basal cell adenoma, cystadenoma, and ductal papilloma, instead show rare cases of recurrence after surgical management (25). Revision surgery implies a higher risk of further surgical complications such as facial nerve impairment; hence, an adequate excision technique is crucial (24). It has been demonstrated that the risk of tumor rupture which leads to tumor recurrence is the same between ECD and conventional superficial parotidectomy (2%–4%) meaning that wider parotid excisions are not a valid contribution to recurrence rate control (26).

**Hematoma and seroma** are not common and in most cases are related to inadequate hemostasis in the intraoperative setting.

## 4 Purpose of the study

Over time, extracapsular dissection (ECD) has replaced, in carefully selected patients, superficial parotidectomy for the treatment of benign parotid gland tumors without significantly increasing recurrence rates (9). This study aimed to evaluate the outcomes of the ECD technique in a series of patients who underwent parotid surgery between January 2019 and December 2021 and compare the results with a cohort of consecutive patients who had undergone parotid surgery by the same surgical team for benign tumors between January 2016 and December 2018, in which superficial parotidectomy technique had been used.

The purpose was to show the overall well-being of patients and the improvement of the quality of their everyday life when superficial parotidectomy was avoided and replaced by a more gland-preserving surgery technique for tumors up to 4.5 cm.

The strategies available range from more limited resections, such as extracapsular dissection (ECD) to more extensive surgical options in various degrees, including superficial partial parotidectomy, partial parotidectomy, deep lobe parotidectomy, and total parotidectomy. While ECD can be performed on small- or medium-sized tumors, total parotidectomy is still reserved and recommended for large or multifocal tumors that involve almost the entire parotid gland (9).

## 5 Materials and methods

The study included a total of 56 patients: 12 females and 16 males in the SP group and 11 females and 17 males in the ECD group. The mean age was comparable, 57.5 years in the first group and 56 years in the second group. The majority of patients in both groups were active smokers or had a previous history of tabagism. This data is shown in Table 1.

**Table 1:** Patients’ variables in the SP and ECD groups.

Variables	SP	ECD
Mean age	57.5 ± 14	56 ± 15
Male (%)	57	61
Female (%)	43	39
Mean tumor size (cm)	3.2 ± 0.73	3.4 ± 0.85
Smokers (%)	54	57

Inclusion criteria for the two cohorts of patients (28 in each group) were non-malignant lesions, tumor size <4.5 cm, primary tumors and non-recurrent ones, and tumor location superficial to the facial nerve. The two cohorts of patients (28 and 28 patients, respectively) were selected based on inclusion criteria such as the maximum tumor size, non-malignancy of the lesion, tumor location, and non-deep lobe tumors. Differences in age and tumor diameter between the two groups were analyzed using the unpaired sample *t*-test. The two-tailed *P*-values were 0.7195 and 0.3491, which did not confirm a statistically significant difference between the two studied groups.

Preoperative evaluation of the non-malignancy of the tumors included ultrasonography, CNB, and CT or MRI. Tumor dimensions were assessed through preoperative imaging, and patients with excessively enlarged tumors were excluded from both cohorts. Regarding tumor location, deep lobe tumors were not included in the study.

**Figure 1:**
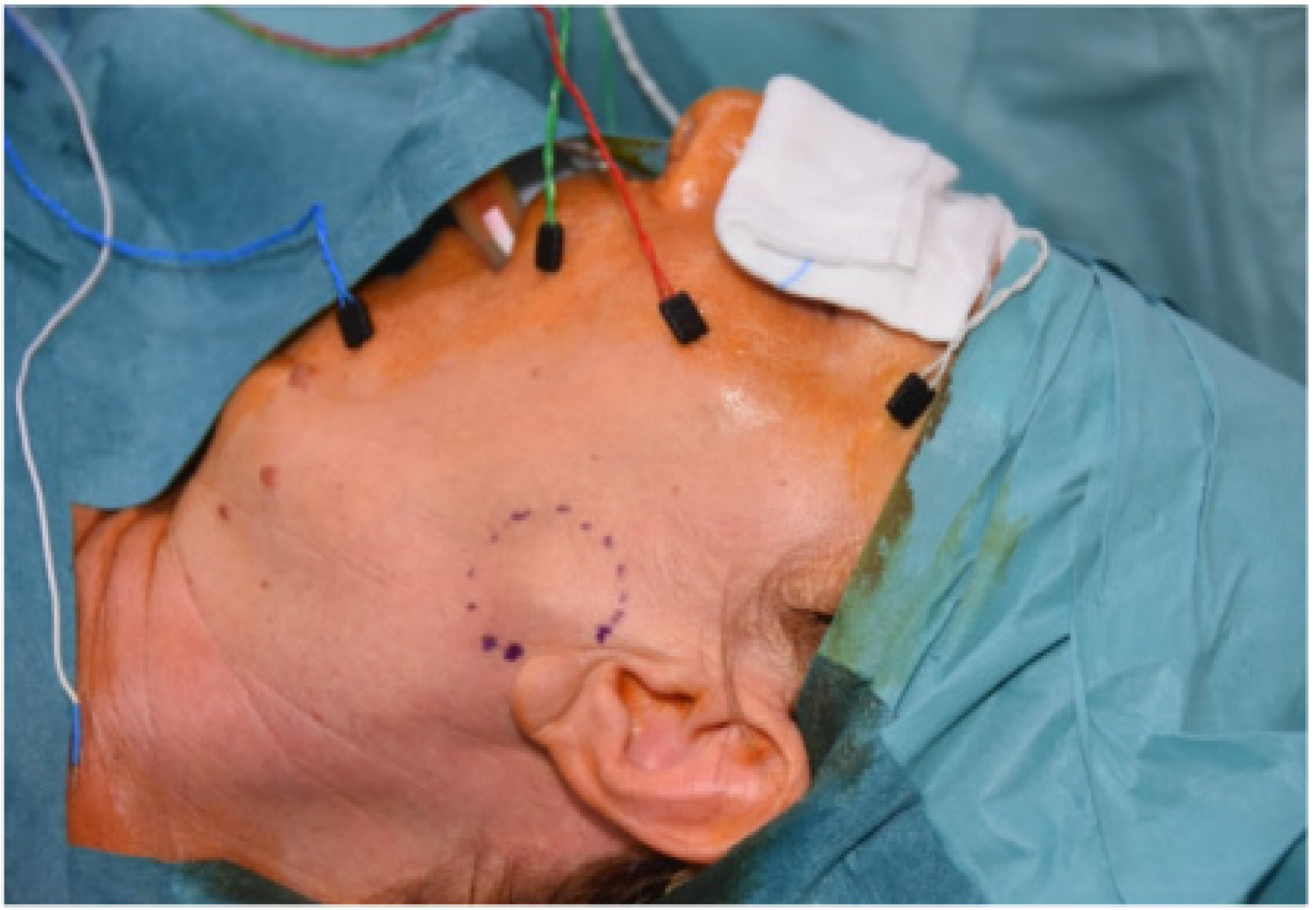
Continuous intraoperative nueromonitoring (cIONM).

**Figure 2:**
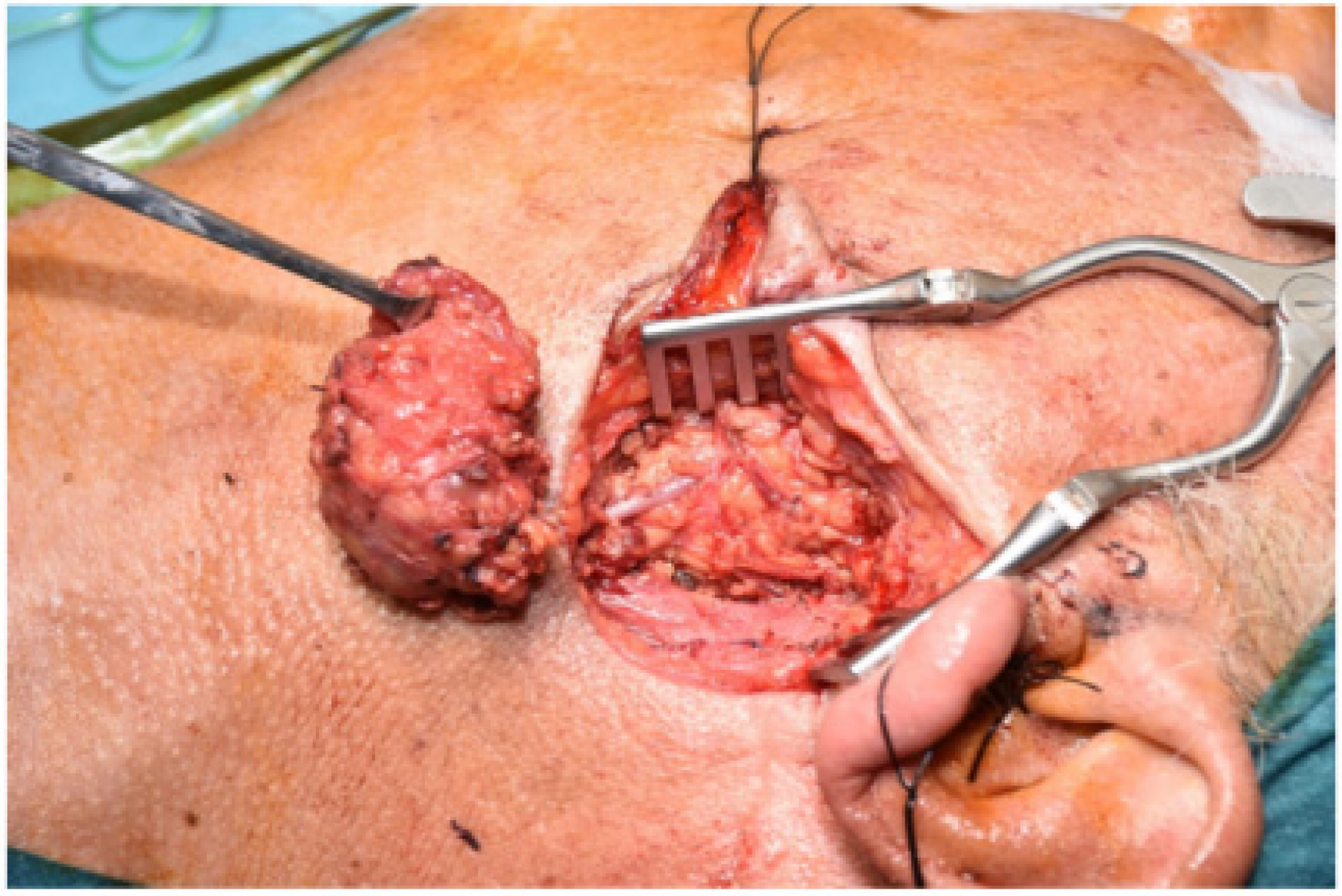
Pleomorphic adenoma excisions.

**Figure 3:**
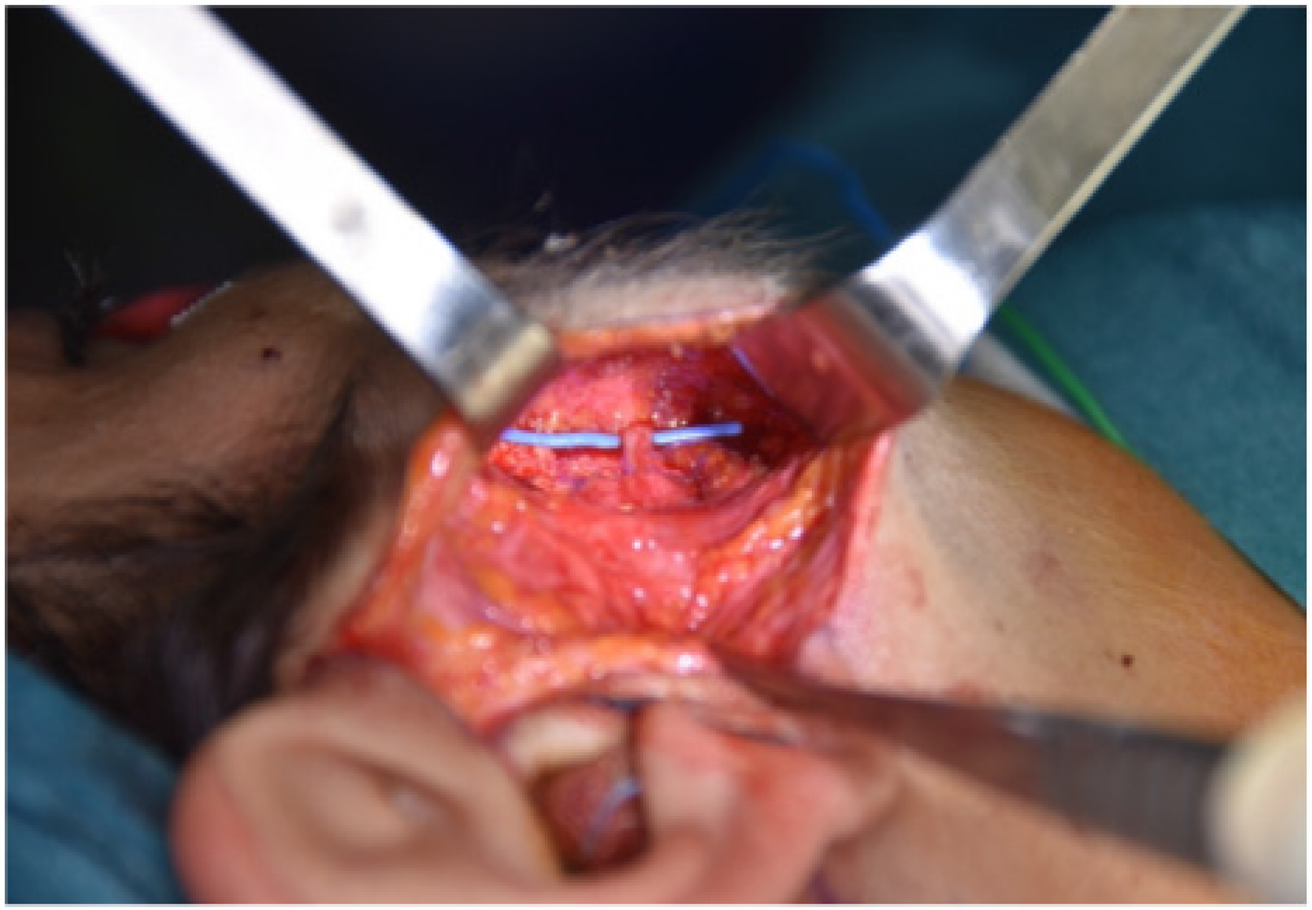
Distal branch of the facial nerve.

All surgeries were performed under Continuous Intraoperative Neuromonitoring (CIONM), the standard of care in head and neck surgery (Figures 1–3). Access incisions were modified Blair incision for the SP group and a shorter preauricular and/or retroauricular incision for the ECD group (Table 2).

**Table 2:** Differences between the SP and ECD groups.

	SP	ECD
Mean incision length (cm)	10.4 ± 0.63	5.5 ± 0.91
Hospital stay (days)	5	3
Mean days with drain	7 ± 1	4 ± 1
Mean follow-up (months)	59.1	21.7

Intraoperative frozen section biopsy (FSB) was performed in all cases suspicious of malignancy. All patients had a suction drain inserted at the end of the surgical procedure and removed when it stopped draining. The time length to removal was a mean of 4.1 days for the ECD and 7.1 days for the SP groups.

In the SP group, histology examination showed the tumors to be pleomorphic adenomas in 9 cases, Warthin's adenomas in 12 cases, oncocytomas in 2 cases, parotid lipomas in 2 cases, papillary ductal adenoma in 2 cases, and monomorphic adenoma in 1 case. In the ECD group, there were 11 cases of pleomorphic adenomas, 13 cases of Warthin's adenomas, 3 cases of canalicular adenomas, and 1 case of oncocytoma. Histological types of both groups are summarized in Table 3.

**Table 3:** Histological types of benign parotid tumors in the two groups.

Histological type	SP	ECD
Warthin's tumor	12	13
Pleomorphic adenoma	9	11
Canalicular adenoma	0	3
Oncocytoma	2	1
Lipoma	2	0
Papillary ductal adenoma	2	0
Basal cell adenoma	1	0

### 5.1 Results

No complications such as infection or wound dehiscence occurred, which would have altered the mean days of hospital stay. There were three cases of injury to the retroauricular nerve in the SP group, one in the ECD. The SP group also had one mild case of Frey's syndrome which resolved after botulinum toxin injection and two cases of salivary fistula which were due to early accidental dislodgement of the drain inserted at the time of surgery. One of these two patients also had a small hematoma which required evacuation. There were no cases of permanent facial palsy in the two groups, but the SP group had three cases of temporary facial nerve weakness which resolved after a few months. Interestingly, there was one case of capsular perforation in the SP group but none in the ECD group, even though the dissection and exposure are much more limited in the latter group. This occurred in a patient with a large dumbbell shaped tumor which extended to a very superficial position and was accidentally nicked during the dissection. This was sutured and spillage was avoided. The ECD group had one case of sialocele which occurred early in our study when it was thought that the small amount of dissection performed did not require postoperative insertion of a drain. The sialocele was aspirated percutaneously and resolved after 2 weeks. The SP group instead had two cases of sialocele. With regard to the aesthetic results of the procedures, facial hollowing to some degree was more frequent in the SP group, and the modified Blair type of incision tended to leave a more visible scar. One patient in the SP group who had the modified Blair incision developed a hypertrophic scar in the posteroinferior ear segment which was treated locally with serial injections of triamcinolone. Fisher's exact test was used to determine any significant difference in the results between the two groups. As for complications compared individually, the test did not demonstrate any meaningful difference due to the limited study sample, but the superiority of ECD procedures compared to SP procedures was shown as far as total complication rates are concerned. In this case, Fisher’s exact test statistic value was 0.0043 (significant at *P* < 0.05) (Table 4).

**Table 4:** Intraoperative and postoperative complication (extended version).

Complication	PSP n.pz	PSP% pz	ECD n.pz	ECD% pz	*P*
Patients	28		28		
Frey's syndrome	1	3.6	0	0	1
Transient FN palsy	3	10.7	0	0	0.4909
Permanent FN palsy	0	0	0	0	1
Salivary fistula	1	3.6	0	0	1
Scialoma	2	7.1	1	3.6	1
Hematoma/seroma	1	3.6	0	0	1
Retroauricolar nerve lesion%	3	10.7	1	3.6	
Capsular perforation	1	3.6	0	0	0.611
Total complication rate	12	42.8	2	7.1	**0.0043**

Bolded values denote significance at *P* < 0.05.

The mean follow-up was 59.1 months in the SP group and 21.7 months in the ECD group. Each patient underwent routine physical examination after 3 months, 6 months, and 1 year. Ultrasonography of the salivary glands was performed after 6 months and 1 year. Long-term follow-up consisted of physical examination and US yearly thereafter. Even though follow-up in our series was too short to properly evaluate the risk of tumor recurrence, there have been, to date, no cases of recurrence in either of the two groups.

## 6 Discussion

Salivary gland tumors represent only 3%–6% of all head and neck neoplasms and are mostly benign. The parotid gland is the most commonly affected salivary gland. The main purpose of surgical treatment for parotid gland tumors is to achieve complete tumor removal while preserving the functional integrity of the facial nerve.

In 1802, Bertrandi was the first surgeon to perform the excision of parotid neoplasms using a demolitive approach, which, in many cases, could lead to damage to the facial nerve and surrounding anatomical structures.

The first to perform the surgical technique used nowadays were Beahrs and Adson in 1958, who identified landmark points to avoid, or at least limitate, nerve injuries and reduce complications associated with salivary gland surgery such as those related to gland manipulation and scarring of surgically treated surfaces (27).

In concordance with common surgical experience, an ever-increasing number of literature reports point toward the remarkable shift of paradigm that has permeated the field of parotid gland surgery in the last 25 years: it is not simply a change in technique, it represents a general change of philosophy toward avoiding iatrogenic injury of the facial nerve (28).

Extracapsular dissection is a minimally invasive technique that follows principles distinct from those of traditional parotid surgery. In traditional parotid surgery, the facial nerve is the focal point of the dissection, and the salivary tissue is carefully separated from its surface.

In contrast, extracapsular dissection is focused on safely parting and dissecting the parotid parenchyma at a small distance from the capsule to expose the tumor (29).

The advantage of not closely dissecting the gland off the nerve has been recently demonstrated in three meta-analyses (viz., Albergotti et al.; Foresta et al.; Xie et al.): extracapsular dissection has been associated with a lower incidence of transient nerve injury, reduced hospital stay, and fewer complications compared to traditional superficial and total parotidectomy, with no increased risk of recurrence (30–32).

Among the cornerstone papers available, the study of Mcgurk et al. examines the approach of the senior author, over the past 20 years, on 97 patients with a discrete parotid lump and FNAC results indicative of a benign tumor. Over half of these tumors were located in the deep lobe. Extracapsular dissection (ECD) and extended ECD were not restricted either by tumor size or site.

The authors reported 9 out of 97 (10.3%) cases of mild facial nerve injury, 4 of which involved patients with low-grade malignant tumors. Excluding these, the facial nerve injury rate was 5 out of 97 (6%), all of which were transient. Other complications included two cases of sialocele, three hematomas, and two instances of first-bite syndrome.

The authors conclude supporting the technique, amenable to all parotid lumps and not restricted by site or size (29).

Among the largest series available in the literature, Thölken et al. included 300 of 579 patients in a prospective study at a university hospital (33). Transient postoperative facial paralysis occurred in 45 patients (15%), while the rate of permanent facial palsy was 3.7% (11 patients). Extracapsular dissection, partial parotidectomy, superficial parotidectomy, and total parotidectomy had median operative time of 87 min (quartiles: 59.0–107.0), 102 min (quartiles: 79.0–136.0), 145 min (quartiles: 121.0–163.5), and 212.5 min (quartiles: 178.8–272.0), respectively: the difference in operative time among the different surgical techniques was found to be significant (*P* < 0.001).

The risk of transient postoperative facial palsy varied across the different surgical techniques (ECD, 5.8%; PP, 29.2%; SP, 20.0%; and TP, 44.1%; *P* < 0.001): it was significantly lower after ECD compared with other surgical techniques.

After 18 months, the risk of facial nerve palsy was significantly lower after ECD than PP or SP (ECD, 0.5%; PP, 12.2%; SP, 11.5%; TP, 2.9%; *P* < 0.001).

In the multivariate logistic regression model, the operative time was found to be an independent risk factor for transient facial palsy (OR = 3.3, 95% CI: 1.7–6.6, *P* < 0.001), while permanent facial palsy was associated with larger tumors (OR = 5.3, 95% CI: 1.1–25.5, *P* = 0.039).

Surgical technique was found to be significantly related to transient facial palsy (95% CI: 2.4–15.9, *P* < 0.001). No other early complications were significantly associated with the type of surgery (all *P* > 0.05).

Within the 18-month timeframe, 9.3% of patients experienced greater auricular nerve dysfunction, and 5.3% developed Frey's syndrome. The surgical technique was significantly associated with the occurrence of Frey's syndrome (*P* = 0.002) and showed a marginally significant association with greater auricular nerve dysfunction (*P* = 0.051) (33).

A recent retrospective study by Mantsopoulos et al. (2024), conducted at an academic tertiary referral center for salivary gland pathologies (Otorhinolaryngology, University of Erlangen-Nuremberg, Germany), examined a total of 4,037 cases of benign parotid neoplasms.

Extracapsular dissection was performed in 2,670 (66.1%), partial superficial parotidectomy in 235 (5.8%), lateral parotidectomy in 334 (8.3%), and total parotidectomy in 798 (19.8%) out of 4,037 cases. Permanent facial nerve palsy occurred in 60 patients (1.4%), Frey's syndrome in 284 patients (7.0%), and salivary gland fistulas in 291 patients (7.2%) out of 4,037 cases. A comparison of various surgical approaches revealed significantly better outcomes for extracapsular dissection compared to non-extracapsular surgeries in terms of facial nerve palsy (*P* < 0.001) and Frey's syndrome (*P* < 0.001).

No statistically significant difference was seen between extracapsular and non-extracapsular surgery concerning the incidence of salivary fistulas (*P* = 0.406) (28).

Over time, extracapsular dissection has been increasingly and successfully used to treat parapharyngeal pleomorphic adenomas, well-defined solitary cystadenolymphomas at the caudal pole, multiple Warthin tumors, and larger, more complex pleomorphic adenomas exhibiting the pattern of pseudopodia and satellite nodules (34–38) lateral to the facial nerve (39). It has also been successfully applied to lesions in the deep lobe, accessed from the caudal side through the “door” of the posterior belly of the digastric muscle (28).

Subsequently, several studies have suggested that carefully selected low-stage, low-grade malignant tumors can be managed exclusively through extracapsular dissection (40–44).

Mantsopoulos et al. reported outstanding oncological outcomes on forty patients with T1–T2 low-grade parotid malignancies.

In the subgroup of R0 patients treated with extracapsular dissection (ECD), the 5-year disease-specific survival was 100%, and local disease control was also 100% (with a mean follow-up of 3.1 years). Significantly, worse functional outcomes were observed in cases that required completion surgery (*P* = 0.006) (40).

The gradual overcoming of the “one-size-fits-all” approach to facial nerve dissection, along with the increased and improved use of extracapsular dissection, has led to a decrease in the more severe complications associated with parotid surgery. Neither the occurrence of salivary fistulas, which could have been promoted by preserving a large amount of parotid parenchyma through the extended application of extracapsular dissection of a lesion, has increased.

The perioperative application of scopolamine (45) prevents the expected increase in the rate of fistulas, a common topic of skeptics and detractors of “gland-preserving surgery.”

Our data on 56 patients clearly shows the benefits of extracapsular dissection gland-sparing surgery (ECD) compared to superficial parotidectomy (SP) when feasible. Shorter hospital stays, lower complication rates, and more aesthetically pleasing results, due to shorter scars and less facial hollowing, constitute the advantages of this procedure over superficial parotidectomy.

Extracapsular dissection of benign parotid tumors may be the ideal treatment, in selected cases, for tumors located in the lateral segment of the parotid gland, superficial to the facial nerve. Among parotid neoplasm, cystadenolymphomas and pleomorphic adenomas retain ideal tumor characteristics: the well-circumscribed, firm capsule and the caudal superficial localization on most of these lesions have allowed for less invasive surgical strategies (46–48).

However, superficial parotidectomy should still be performed for tumors larger than 4.5 cm in diameter or located in the medial segment of the parotid gland, deep to the facial nerve. ECD should be applied in properly selected cases and further prospective studies may clarify the optimal indications.

### 6.1 Recurrence

After surgical resection, a small percentage (2%–5%) of benign parotid neoplasms may recur: recurrence is a challenging issue (49). Iatrogenic tumor puncture, cell spillage following rupture, or violation of the capsule during surgery have been advocated as major risk factors. Other contributing factors include advanced age, deep parotid lobe location, larger tumor size, and tumor adhesions to the facial nerve (50).

Pleomorphic adenomas are inclined to recurrence and are suspected to slowly gain malignant behavior after multiple recurrences. Myxoid subtypes, in particular, often demonstrate thinner and incomplete capsules, making them more prone to recurrence: larger tumors not only tend to have incomplete capsules but are also associated with a higher number of satellite nodules (51–54).

Reoperation, which is technically demanding, carries a high risk of facial nerve (FN) injury. The likelihood of permanent FN damage rises with each successive surgery. Factors that complicate surgery for recurrence include the dissection through a scarred tumor bed and the tendency for multifocal disease (55, 56).

The role of radiation therapy (RT) in treating recurrent parotid neoplasms remains controversial. There is a lack of well-designed studies that clarify the potential benefits of radiotherapy as an adjuvant treatment in patients where complete excision is not feasible, in cases with close surgical margins, intraoperative tumor spillage or capsule violation, less favorable histological features, or multiple recurrences with multifocal disease (55–58).

Although some studies have not shown a significant improvement in tumor control rates after postoperative RT, other authors argue that RT may provide better local control than surgery alone.

Chen et al. assessed the role of RT in managing recurrent disease and reported a 94% local control rate over 20 years in a series of 34 patients. However, one patient developed a second malignancy about 14 years after treatment (57).

Many authors, particularly surgeons, are cautious about using radiation therapy (RT) due to its potential side effects and the risk of RT-induced malignancies, especially in younger patients (59, 60). Furthermore, there is a lack of prospective studies comparing the outcomes of adjuvant RT with surgery alone (60).

## 7 Conclusions

The choice of extracapsular dissection in select cases aims to ensure patient safety, preserve functional outcomes, and minimize postoperative surgical complications. This change in approach has significantly improved the postoperative quality of life for patients. Extracapsular dissection can be described as a tailored, electromyographic-controlled dissection procedure, performed around the tumor without relying on specific anatomic landmarks. Since the choice of surgical approach is often made intraoperatively and it is not always possible to accurately assess the tumor’s proximity to the facial nerve before surgery, less invasive and more conservative techniques (61–69) should be performed by experienced surgeons who can identify and dissect the facial nerve when necessary.
